# Investigation on Effect of Material Hardness in High Speed CNC End Milling Process

**DOI:** 10.1155/2015/762604

**Published:** 2015-12-31

**Authors:** N. V. Dhandapani, V. S. Thangarasu, G. Sureshkannan

**Affiliations:** ^1^Faculty of Mechanical Engineering, Karpagam College of Engineering, Coimbatore 641032, India; ^2^Faculty of Mechanical Engineering, Nehru Institute of Engineering and Technology, Coimbatore 641105, India; ^3^Faculty of Mechanical Engineering, Coimbatore Institute of Technology, Coimbatore 641014, India

## Abstract

This research paper analyzes the effects of material properties on surface roughness, material removal rate, and tool wear on high speed CNC end milling process with various ferrous and nonferrous materials. The challenge of material specific decision on the process parameters of spindle speed, feed rate, depth of cut, coolant flow rate, cutting tool material, and type of coating for the cutting tool for required quality and quantity of production is addressed. Generally, decision made by the operator on floor is based on suggested values of the tool manufacturer or by trial and error method. This paper describes effect of various parameters on the surface roughness characteristics of the precision machining part. The prediction method suggested is based on various experimental analysis of parameters in different compositions of input conditions which would benefit the industry on standardization of high speed CNC end milling processes. The results show a basis for selection of parameters to get better results of surface roughness values as predicted by the case study results.

## 1. Introduction

High speed machining is being considered as one of the fast developing applications in machining of high precision surfaces of components. The past decade is known for development of importance for CNC machines, and CNCs started replacing many special machines. Recent studies show that finish machining of precision industrial components needs to be manufactured with high standards of accuracy and tolerance design for high precision applications. Eventually by the introduction of high speed machining, the requirement for finish machining becomes meager by the capability of modern CNC machines that are producing parts with very low surface roughness and high levels of geometric accuracies. This research is based on extensive case studies conducted for small and medium size precision products manufacturing companies around Coimbatore. The experiments were conducted on a standard 4-axis high speed milling machine, and the results were compared for the desired parameters to responses also and we analyzed the effects of different parametric selection criteria to minimize the human intervention in parameter selection.

## 2. Related Research

The recent research in this area by Aggarwal and Singh [1] developed mechanistic model similar to dynamic model by Quiza Sardiñas et al. [2], and statistical artificial intelligence models and also two-phased optimization method by Tzeng and Chen [3] are some examples of cutting force modeling and optimization approach using parameter design. Zolfaghariy and Liang [4] addressed dynamic analysis of chip formation, cutting temperatures, tool stresses, and cutting forces. Many researches inferred that the effect of cutting forces developed in milling process can be directly used to estimate process performances of tool wear, cutting time, surface finish, and so forth. Studies conducted by Kadirgama and Noor [5] showed that peak cutting force component in the feed direction is more sensitive to the tool wear indicator of the flank wear. The importance of tool wear monitoring and optimization method brought out by Lou et al. [6], Fang et al. [7], and Aggarwal and Singh [1] emphasized more on cutting force measurement as one of the most common parameters for online tool monitoring, but the theoretical study also highlighted that the maximum durability of cutting tool is 90 mins; then the tool is eventually replaced by new one. The recent research by Kechagias et al. [8] investigated tool wear in high speed machining process with new and worn-out milling cutters. The cutting force by the tool is directly affecting the tool life but the case studies conducted by us in SMEs of CNC class were able to survive the expectations of surface roughness. The material removal rate is another criterion which summarizes the productivity of the process. The CVD coated end milling inserts are not affecting the surface finish levels up to 90 minutes of operation. Hence it is to be noted that during this period surface roughness is directly influenced only by cutting parameters. Proper selection of cutting parameters would result in high quality parts and greater savings in production time and production cost within first 90 minutes of operation.

## 3. Parameters and Responses

The spindle speed, feed rate per revolution, depth of cut, cutting force exerted, cutting tool material and coating, hardness and machinability of material, vibration on tool, and amount of coolant flow were taken as the input parameters of the process and the tool wear rate, average surface roughness, and material removal rate are the respective responses for determination of expected output for the process as listed in Table 1. An extensive list of materials in the ferrous and nonferrous category is shown in Table 2. The materials for the study were taken on the basis of frequency of orders to these companies, different hardness values, and machinability factors. The parameter selection is based on case study at small and medium type enterprises around Coimbatore for high speed machining job order type production houses for precision parts. An example set of factors and levels are listed in Table 1 for the stainless steel material; for softer materials the value of depth of cut increases up to 3.0 mm.

Effects of hardness, ductility, and brittle fractures were also compared for the output characteristics requirements of surface roughness values and material removal rate. The spindle speed once increased from lower limiting value, the evidence of reduction in surface roughness is recorded, which promotes the use of high speed machining for the precision component manufacturing. Similarly all the factors have their influential effects on the final outcomes like surface roughness and material removal rate.

## 4. Experimental Method

Detailed experimental analysis has been carried out based on standardized DOE of L27 array of experiments to study and optimize the processing parameters. Surface roughness and material removal rate in a high speed milling process analyzed properly based on the preliminary trials on selected independent process parameters that affects the surface finish and material removal rate were identified as spindle speed (*A*), feed rate (*B*), depth of cut (*C*), and type of insert (*D*), namely flat, bull, and ball nose inserts. One of the materials, Alloy Steel EN24 material, is machined with DOE set of trials using its ranges of operation as per the recommendations of machine tool manufacturer and is presented and similar experiments for all 7 materials were performed and brought out results as listed in Table 5.

The average surface roughness (*R*_*a*_) was measured after machining, in the feed direction using a Mitutoyo roughness measuring instrument (SJ-210 Series). Measuring parameters as listed and studied include the average (*R*_*a*_) and the material removal rate. The spindle speed (*A*) in rpm and feed per tooth (*B*) mm/rev/tooth, depth of cut (*C*), and type of insert (*D*) used are taken as variables. An array of L27 based on DOE is considered to conduct the experiments on standardized equipment (DECKEL MAHO 64V Linear, a 4-axis milling center) and used for the study and with the parameters and ranges. The above experiments were performed with a CVD coated tungsten carbide tool at 27 different combinations of input parameters under full coolant supply conditions. The effects of the other process parameters on the surface roughness like tool wear rate, built-up edge size, chip curl radius, and chip thickness are also accounted in determining the surface roughness up to 90 minutes of operation of cutting tool without considerable wear and found not affecting responses.

The responses for the same variables are measured by Mitutoyo surface gauge and compared for the correctness. Material removal rate is also computed using approximation method for comparison as the MRR is the indicator of productivity but has more factors but the tangible one accounts for comparison. The bar material is initially prepared using the conventional milling machine for the required dimensions 120 × 50 × 15 mm and surface is finished up to 0.02 mm accuracy in a standard surface grinding machine. Coordinate measuring machine (CMM) is used to check dimensional accuracy and geometrical requirements of the test piece. The responses surface roughness (*R*_*a*_) and material removal rate (MRR) in end milling for various materials were computed based on the Box-Behnken response surface methodology (RSM) for four-factor (spindle speed, feed rate, depth of cut, and type of material on hardness) three-level orthogonal experiments. An orthogonal array of L27 is chosen to experiment and analysis of variance (ANOVA) is used to obtain the objective function for optimization of initial basic feasible results.

## 5. Optimization Using Response Surface Method

Table 3 shows that the input factors are equally vital and need to be given equal importance as this work is a 4-factor three-level factorial technique which was employed for the development of design matrix to conduct the required experiments. The surface quality of machined parts is the value of surface roughness or the waviness which is mainly decided by the factors cutting speed, feed, and depth of cut and type of milling cutter beyond the levels of influence by other factors. The criterion for optimization, *R*_*a*_, has to be minimized and MRR is to be maximized but they are contradictory in nature; while getting a trade-off function between these two responses the priority is fixed as *R*_*a*_ minimization has to be given first priority.

The actual variables in their natural units of measurement are used in the experiment; however, we used our coded variables while designing experiment, *x*_1_ and *x*_2_, which will be centered on 0 and extend +1 and −1 from the center of the region of experimentation. Therefore, the natural units were taken and then centered and we rescaled them to the range from −1 to +1, using best prior solution, and searched for the optimum spot where the response is either maximized or minimized.

Then fitting with second-order model, which is a second-order quadratic terms, consider

(1) y=β0+β1x1+β2x2+β12x1x2+β11x12+β22x22+ε.

Box Behnken based RSM is desirable since there are more points in the middle of the range and they are not as extreme. The Box-Behnken is most suited for the points that are not as extreme as all of the factors, where *b*_0_, *b*_1_, and so forth are the estimated parameters.

The studies reported in literature mostly concentrated on the centre line average roughness *R*_*a*_ value for surface quality and this study in particular permits the user to choose the appropriate requirement either *R*_*a*_ or MRR to a particular operation. By L27 array, the correlation equation obtained for the required responses and numbers of runs were conducted for ductile and brittle materials based on the derived correlation equation. The measured values of the responses with required input cutting parameters were analyzed through Box-Behnken response surface methodology. The predictions based on the Box-Behnken method are compared with the actual values of surface roughness and it is shown that the prediction is closer to the originally measured values of the responses against the variations of input process parameters of CNC high speed machining process. The same may be taken as the pilot study into the very high speed machining processes and could be used for simulation process to predict the surface roughness values of end milling process.

### 5.1. Analysis of Results

The optimal cutting parameters were obtained using the objectives as to minimize the surface roughness and simultaneously maximize the material removal rate, within the specified limits of parameters as maximum and minimum boundary conditions for optimization. Requisite quadratic equations for surface roughness and material removal rate were established using ANOVA and statistical correlation analysis by design expert software gives a nonlinear model that best fits the variation of cutting parameters and describes the variation of surface roughness and material removal rate. A response surface is developed to predict surface roughness (*R*_*a*_) and material removal rate (volume/sec) and set of cutting parameters is selected for a given range of material removal rate until the tool wear reaches 0.6 *μ*m, by which the surface roughness is not affected. Complex materials for aerospace and automotive industries require tough conditions for high precision requirements of lesser tool wear, low distortion and deflection, reliable and repeatable accuracies, good surface finish, and burr-free edges which are key of high speed end milling processes; hence they can be used for high productivity without sacrificing the accuracy.

### 5.2. ANOVA for Response-1 Surface Roughness (*R*_*a*_)

The Model *F*-Value (Table 4) of 17.27 implies that the model is significant. There is only a 0.01% chance that a “Model *F*-Value” this large could occur due to noise. Values of “Prob. > *F*” less than 0.0500 indicate that model terms are significant. In this case *A*, *B*, *D*, *AD*, *BC*, *BD*, *D* ^2^ are significant model terms. Values greater than 0.1000 indicate that the model terms are not significant, if there are many insignificant model terms (not counting those required to support hierarchy). It has been pointed out that an increase in tool life would lead to reduction in production cost, whereas increase in productivity would lead to reduction in production cost. Greater gains are definitely possible by increasing metal removal. Hence, there is a necessity to develop adequate models for end milling process to achieve the benefits like productivity and quality. The objectives for roughing and finishing are quite different; volume of material to be removed is the output target in rough end milling and the good surface finish and dimensional accuracy are the vital factors in finish end milling.

### 5.3. Prediction Equation for *R*_*a*_ in terms of Actual Factors

Consider

(2) Minimize  Ra=+3.37579−2.27840E−004A−7.09722E−004B+0.66667C−2.08583D−6.6E−008AB−1.55556E−004AC+1.08889E−004AD+2.36111E−003BC+6.04167E−004BD+0.46667CD+1.07202E−008A2+1.20370E−007B2−2.11574C2−0.66917D2.

### 5.4. ANOVA for Response-2 Material Removal Rate

The Model *F*-Value (Table 5) of 3.65 implies that the model is significant. There is only a 1.05% chance that a “Model *F*-Value” this large could occur due to noise. Values of “Prob. > *F*” less than 0.0500 indicate that model terms are significant. In this case *A*, *C*, *AD*, *D* ^2^ are significant model terms. Values greater than 0.1000 indicate that the model terms are not significant. If there are many insignificant model terms (not counting those required to support hierarchy), model reduction may improve your model.

### 5.5. Prediction Equation in terms of Actual Factors

Consider

(3) Maximize  MRR=−75.62243−0.15877A+1.23153B−1587.73555C+1125.06856D−5.26098E−005AB+0.24438AC−0.12325AD−0.78345BC−0.42108BD+534.25965CD+1.27055E−005A2−2.47969E−004B2+1574.58562C2+469.23425D2.

The high speed milling has become a costly process because of high investment machine tool and cutters made out of tungsten carbides, polycrystalline diamond (PCD), and polycrystalline cubic boron nitride (PCBN). Hence improper selection of cutting conditions would result in excessive tool wear that may damage part profiles and surface finish, when high degree of surface finish (0.05 to 0.4 *μ*m) is essential for high precision parts. Generally machine parts require surface finish in the range of 0.8 to 3.2 *μ*m. Literature shows that high speed end milling is capable of producing the surface finish of 0.2–0.8 *μ*m.

**Figure 1:**
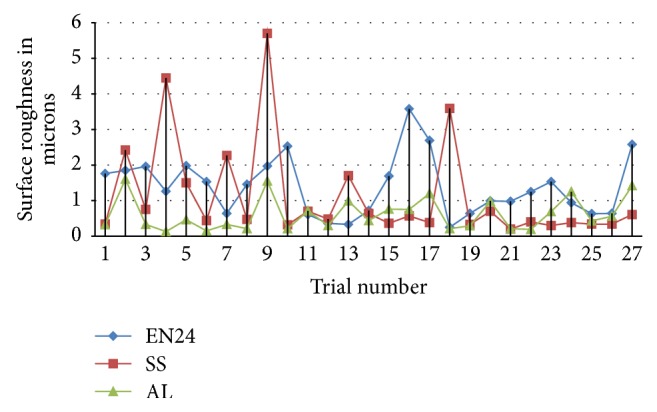
Surface roughness variations of ductile materials in microns.

**Figure 2:**
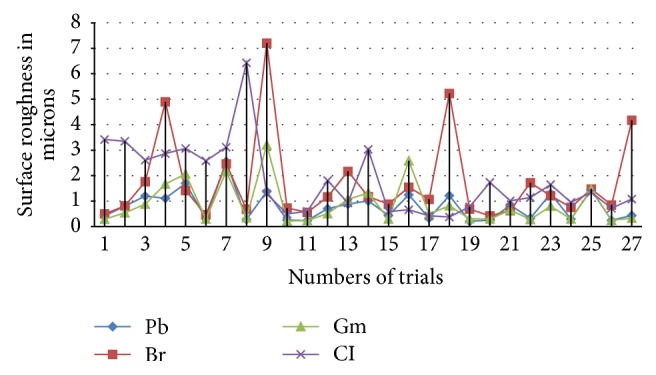
Surface roughness variations of brittle materials in microns.

Surface roughness values obtained out of different ductile and brittle materials were plotted in Figures 1 and 2. These graphs show that there is considerable deviation of roughness values between materials but they are lying in line with their hardness values of 92, 180, and 280 and the pattern of dispersion also follows the same. The harder the material in ductile class, the higher the surface roughness values, but in brittle materials it was identified that closer variation between them and the porous nature play major role in machinability of brittle materials and most porous material recorded with higher roughness values due to its microstructure.

**Figure 3:**
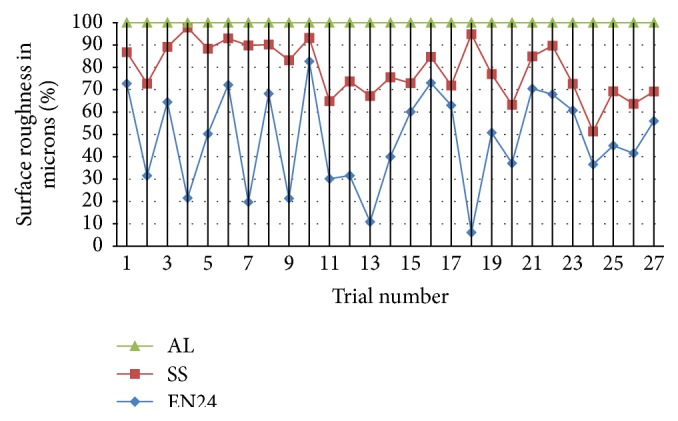
Comparison percentile graph (taking highest as 100%) for surface roughness variation of ductile materials.

**Figure 4:**
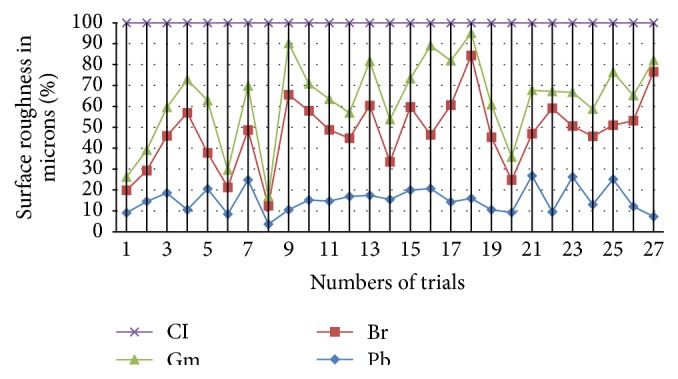
Comparison percentile graph (taking highest as 100%) for surface roughness variation of brittle materials.

Figures 3 and 4 show the variation in percentile graph for better understanding of the different behaviors of materials in respective classes. The responses of surface roughness are plotted to give better information for the analysis.  Figure 4 gives out the details of brittle fracture based materials in percentile graph for the better understanding of the variation between the material behaviors.

Table 6 indicates various optimality values derived from the response surface methodology optimization method, some of the optimum runs ranging from 58 to 169 runs depending upon the optimum value of desired value of 0.2-micron finish roughness. One could easily understand that the ductile materials behave as natural linear variant to hardness; that is, parameter values reduce as the hardness increases; the hardness hence is taken as defining factor for ductile materials but for the brittle materials it should be noted that these values are more dependent on the porosity and density values; more porous material gets better machinability but generally erodes the cutting tool easily and is recorded 2.65 times more than that of ductile materials. This phenomenon needs to be investigated further to improve the robustness of the research. When the machinability is desired, then the wide array of events to be studied would also be more like tool wear rate, microstructure, work hardening effects, and many more in the future research.

## 6. Conclusion

The high speed CNC machining is a vital and costly machining process and a less harder material in ductile class would yield good surface finish whereas the closer variation in brittle class, but less porous brittle material, has good surface finish with higher depth of cut and feed rate and spindle speed more than the mid value, which improves productivity.

- The results show that there is more concentration around the band of 0.26–0.25-micron finish in the levels of spindle speed as in the range of 11000 to 12000 rpm at the feed rate of 600 to 650 m/min and between 0.66 mm and 0.87 mm of depth of cut.
- Hence it is to be noted that the midpoint values may yield good results for various ductile and brittle materials. All these values vary with respect to material hardness and follow a pattern for ductile and brittle materials.
- By adopting this technique, the optimum performance is ensured in the SMEs in the utilization of the resources for production, thereby improving the productivity of the firm. Efforts are being made to simulate and verify the same with very high speed CNC machining and also to incorporate more materials, thereby giving the SMEs better decision making tool for machining optimization solutions.

## Figures and Tables

**Table 1:** Factor and levels for testing for stainless steel.

Factor	Level 1	Level 2	Level 3
Cutting speed (rpm)	4000	9000	12000
Feed rate (mm/rev/min)	900	1800	3000
Depth of cut (mm)	0.1	0.2	0.3

**Table 2:** Defining properties on machinability of materials.

Sl no.	Material	Density Kg/M^3^	Hardness BHN	Type
1	Brass	8500	20	Brittle
2	Gunmetal	8719	60	
3	Phosphor bronze	8900	160	
4	Cast iron	7800	415	

5	Aluminium	2700	90	Ductile
6	Stainless steel	8000	196	
7	Alloy Steel EN24	7840	280	

**Table 3:** DOE trials OF L27.

Run	Speed (rpm)	Feed (mm/rev/min)	Depth of cut (mm)
1	1	1	1
2	1	1	2
3	1	1	3
4	1	2	1
5	1	2	2
6	1	2	3
7	1	3	1
8	1	3	2
9	1	3	3
10	2	1	1
11	2	1	2
12	2	1	3
13	2	2	1
14	2	2	2
15	2	2	3
16	2	3	1
17	2	3	2
18	2	3	3
19	3	1	1
20	3	1	2
21	3	1	3
22	3	2	1
23	3	2	2
24	3	2	3
25	3	3	1
26	3	3	2
27	3	3	3

**Table 4:** ANOVA for Response-1 surface roughness (*R*_*a*_).

Source	Sum of squares	df.	Mean square	*F*-value	*P* value Prob. > *F*
Model	22.50	14	1.61	17.27	<0.0001
*A*, speed	14.04	1	14.04	150.91	<0.0001
*B*, feed	1.06	1	1.06	11.42	0.0045
*C*, DoC	0.046	1	0.046	0.49	0.4952
*D*, insert type	0.84	1	0.84	9.00	0.0095
*AB*	0.13	1	0.13	1.39	0.2576
*AC*	0.18	1	0.18	1.90	0.1901
*AD*	0.96	1	0.96	10.32	0.0063
*BC*	0.72	1	0.72	7.77	0.0146
*BD*	0.53	1	0.53	5.65	0.0323
*CD*	0.078	1	0.078	0.84	0.3742
*A* ^2^	0.31	1	0.31	3.29	0.0914
*B* ^2^	0.012	1	0.012	0.13	0.7229
*C* ^2^	0.24	1	0.24	2.53	0.1342
*D* ^2^	2.90	1	2.90	31.22	<0.0001

**Table 5:** ANOVA for Response-2 material removal rate.

Source	Sum of squares	df.	Mean square	*F*-value	*P* value Prob. > *F*
Model	9.114*E* + 006	14	6.510*E* + 005	3.65	0.0105
*A*, speed	3.231*E* + 006	1	3.231*E* + 006	18.13	0.0008
*B*, feed	2.250*E* + 005	1	2.250*E* + 005	1.26	0.2800
*C*, DoC	1.541*E* + 006	1	1.541*E* + 006	8.65	0.0107
*D*, in. type	3055.65	1	3055.65	0.017	0.8977
*AB*	80708.65	1	80708.65	0.45	0.5119
*AC*	4.354*E* + 005	1	4.354*E* + 005	2.44	0.1403
*AD*	1.230*E* + 006	1	1.230*E* + 006	6.91	0.0199
*BC*	79547.84	1	79547.84	0.45	0.5149
*BD*	2.553*E* + 005	1	2.553*E* + 005	1.43	0.2512
*CD*	1.028*E* + 005	1	1.028*E* + 005	0.58	0.4602
*A* ^2^	4.294*E* + 005	1	4.294*E* + 005	2.41	0.1429
*B* ^2^	51690.33	1	51690.33	0.29	0.5986
*C* ^2^	1.303*E* + 005	1	1.303*E* + 005	0.73	0.4069
*D* ^2^	1.428*E* + 006	1	1.428*E* + 006	8.01	0.0133

**Table 6:** Results for comparison.

Optimum parameter values			
Hardness	Speed	Feed rate	Depth of cut
Brass (20)	11853	1268	2.34
Gunmetal (60)	11582	2176	2.85
Phosphor bronze (160)	10968	1869	2.78
CI415	9258	1569	2.98
Aluminium (90)	11620	2690	2.96
Stainless steel (196)	10580	2169	0.852
Alloy steel (280)	9864	1985	0.742
